# Haematology and plasma biochemistry reference intervals of Galapagos tortoises from Isabela Island

**DOI:** 10.1093/conphys/coaf054

**Published:** 2025-07-29

**Authors:** Ainoa Nieto-Claudín, Jamie L Palmer, Maris Brenn-White, Fernando Esperón, Santiago Cano, Sharon L Deem

**Affiliations:** Charles Darwin Research Station, Charles Darwin Foundation, Charles Darwin Av, Santa Cruz 200350, Galapagos Islands, Ecuador; Saint Louis Zoo Institute for Conservation Medicine and WildCare Institute Center for Chelonian Conservation, One Government Drive, Saint Louis, MO 63110, USA; Saint Louis Zoo Institute for Conservation Medicine and WildCare Institute Center for Chelonian Conservation, One Government Drive, Saint Louis, MO 63110, USA; Saint Louis Zoo Institute for Conservation Medicine and WildCare Institute Center for Chelonian Conservation, One Government Drive, Saint Louis, MO 63110, USA; Santa Cruz County Animal Services Authority, 1001 Rodriguez Street, Santa Cruz, CA 95062, USA; CISA-INIA-CSIC, Algete-El Casar Road, 28130 Valdeolmos, Madrid, Spain; Veterinary Department, School of Biomedical and Health Sciences, Universidad Europea de Madrid, Tajo Street s/n, 28670 Villaviciosa de Odón, Madrid, Spain; Veterinary Faculty, Complutense University of Madrid, Puerta de Hierro Av, 28040 Madrid, Spain; Charles Darwin Research Station, Charles Darwin Foundation, Charles Darwin Av, Santa Cruz 200350, Galapagos Islands, Ecuador; Saint Louis Zoo Institute for Conservation Medicine and WildCare Institute Center for Chelonian Conservation, One Government Drive, Saint Louis, MO 63110, USA

**Keywords:** Blood parameters, chelonians*, Chelonoidis* spp, reference values, wildlife health surveillance

## Abstract

Wildlife health assessments including haematology and biochemistry parameters are essential to evaluating the well-being of free-living species. In Galapagos, the iconic giant tortoises still thrive in the archipelago despite anthropogenic pressures, with up to 13 extant species distributed across most islands and ecosystems. In previous work conducted by our research group, we described for the first-time reference intervals of haematology and plasma biochemistry in four Galapagos tortoise species. With the aim of continuing to provide cutting-edge health data for Galapagos tortoises, here we report haematology and plasma biochemistry descriptive statistics, reference intervals and cell morphology of tortoises from four different tortoise populations (i.e. Alcedo Volcano, Cerro Azul Volcano, Cinco Cerros and Sierra Negra Volcano)*.* Additionally, we compared values between sexes and applied a principal component analysis to explore differences in haematology and biochemistry parameters between tortoise populations, including those previously published by our research group. Females presented higher calcium, phosphorus and albumin, consistent with vitellogenesis, whereas males had higher packed cell volume and sodium than females. Blood cell morphology was consistent across species. The two main principal components of the multivariate statistical model were unable to explain >44.9% of the variance across tortoise populations. We suggest additional research to explore the correlation between anthropogenic factors (i.e. climate change, pesticides, plastics) and blood values, for a deeper understanding of tortoise physiology and ultimately improved diagnostics and management actions. In the anthropogenic era, understanding the health status of bioindicator species like Galapagos tortoises is mandatory to inform current and future conservation priorities and actions.

## Introduction

Wildlife health assessments are instrumental to understanding the health of free-living individuals and their populations, as well as informing clinical decisions for wild animals under human care (Deem *et al*., 2001). Monitoring haematological and biochemical parameters is an essential component of health evaluations, however, the lack of baseline data for comparison in most wildlife species is a major obstacle to interpreting results (Lewbart *et al*., 2015). Research on reptile health has become urgent in recent years as a consequence of increased removal from the wild for pet ownership and degradation of natural environments; both of which facilitate increases in disease incidence (Stacy *et al*., 2011). As our knowledge of reptile diseases increases, we also realize the importance of obtaining information on the baseline health parameters to understand the health status of individuals and populations (Rivera *et al*., 2009; Nieto-Claudin *et al*., 2022).

In the Galapagos Archipelago, several unique reptile species have survived human predation and currently coexist with a profitable tourist industry and an increased human population (Benitez-Capistros *et al*., 2019; Cajiao *et al*., 2020). Health information on endemic Galapagos reptiles is largely lacking although projects such as the Galapagos Tortoise Movement Ecology Programme (GTMEP) have worked over the last decade to close this gap (Blake *et al*., 2015; Sheldon *et al*., 2016; Nieto-Claudin *et al*., 2021a; Ramon-Gomez *et al*., 2023; Nieto-Claudín *et al*., 2024). The Galapagos giant tortoise is one of the most iconic reptiles of the archipelago, with 16 species described and up to 13 still surviving on the islands according to recent studies (Gaughran et al., 2024). More than 60 years of captive breeding and restoration efforts have resulted in a significant increase in tortoise populations (Cayot, 2021a, 2021b); however, several challenges associated with anthropogenic impacts persist. Current threats to the conservation of these giants include invasive species, habitat degradation, disease, climate change, pollution, poaching and illegal trade (Ellis-Soto *et al*., 2017; Nieto-Claudin *et al*., 2019; Deem *et al*., 2023; Ramon-Gomez *et al*., 2023; Blake *et al*., 2024).

In 2021, our research group described robust haematology and biochemistry reference intervals (RI) in free-living Western Santa Cruz giant tortoises (*Chelonoidis porteri*) (Nieto-Claudín *et al*., 2021b) and more recently, RI of free-living tortoises from Eastern Santa Cruz (*Chelonoidis donfaustoi*), Española (*Chelonoidis hoodensis*) and San Cristóbal (*Chelonoidis chathamensis*) Islands (Nieto-Claudín *et al*., 2024).

With the aim of providing detailed haematology and biochemistry information for all Galapagos tortoise species, here we collected and analysed blood samples from tortoises in southwestern Isabela Island and computed descriptive statistics and RI of tortoise populations of Alcedo Volcano (*Chelonoidis vandenburghi*), Sierra Negra Volcano (*Chelonoidis guntheri*), Cerro Azul Volcano (*Chelonoidis vicina*) and the population from Cinco Cerros area where *C. guntheri* and *C. vicina* (as well as potential hybrids) may coexist.

## Materials and Methods

### Study site

This study was conducted on Isabela Island, as part of a long-term health assessment within the GTMEP and in collaboration with the Galapagos National Park Directorate (GNPD). Isabela Island (S00.9492010°, W090.9704880°) is the largest of the archipelago and each of its five volcanoes hosts a different species of giant tortoise (Tapia, 2021). For the present study, we conducted health assessments on tortoise populations from three of the five volcanoes: Alcedo, Sierra Negra and Cerro Azul.

Alcedo Volcano, located in the middle of the island (S00.4409454°, W091.1068907°), holds the largest number of tortoises, with an estimated 12 000–15 000 individuals of *C. vandenburghi*, according to the most recent census (Tapia, 2021). This species is considered Vulnerable (IUCN, 2023), although its population has been successfully recovering since the eradication of invasive species in 2006. South of Alcedo, the Sierra Negra Volcano (S00.8248506°, W091.1701717°), with the largest crater of the archipelago, is home to the critically endangered *C. guntheri* (IUCN, 2023). This species occurs in distinct subpopulations naturally fragmented by lava fields, on the southern, eastern and western flanks of the volcano. *Chelonoidis guntheri* was the most heavily exploited of all Galapagos tortoise species for oil in the first half of the 1900s, with the current population estimated at 700 individuals according to the latest GNPD census (Gibbs, 2024). Unfortunately, poaching continues to decimate their populations and trafficking of juvenile tortoises has also become a problem in recent years (Gibbs *et al*., 2020). Cerro Azul Volcano, located in the southwest end of Isabela (S00.929378°, W091.37500°), is home to the endangered *C. vicina* tortoise species. Subpopulations are scattered on the flanks of the volcano and along the western coast. They are isolated from *C. guntheri* on adjacent Sierra Negra Volcano by a single lava flow that nearly bisects the island. The latest GNDP census conducted on southern Isabela estimates its population at 5275 tortoises (Gibbs, 2024). Historical exploitation by whaling operations stripped *C. vicina* tortoises from coastal areas where even today poaching by residents occasionally continues (Gibbs *et al*., 2020). Despite ongoing management programmes, human predation and introduced species such as feral pigs, cattle, dogs, fire ants, guava and blackberry continue to threaten these already decimated populations along southern Isabela (Gibbs *et al*., 2020). Cinco Cerros (S01.009200°, W091.238004°) is the only location where Sierra Negra (*C. vicina*) and Cerro Azul (*C. guntheri*) tortoises may coexist through a small opening in the lava along the southern coast (Gibbs *et al*., 2020). It is also home to an unusual subpopulation of tortoises with a very special morphology; ‘crushed tortoises’, directly translated from the Spanish word ‘aplastadas’. While morphologically distinct, these ‘flat’ tortoises are genetically related to *C. guntheri* (Gibbs *et al*., 2020).

**Figure 1:**
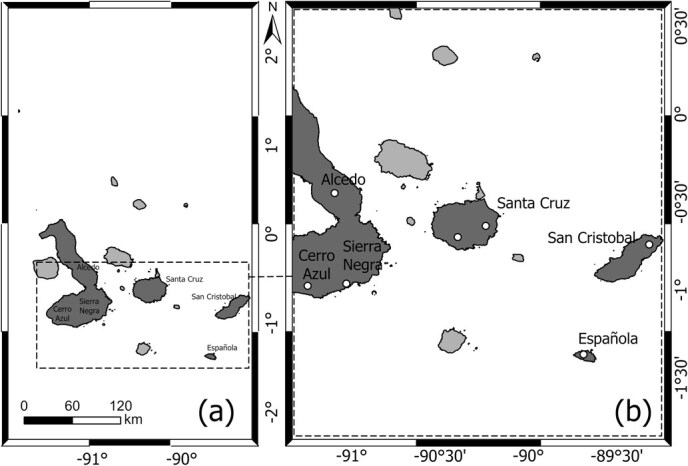
Sampling locations of Alcedo Volcano giant tortoises (*C. vandenburghi)*, Sierra Negra Volcano tortoises (*C. guntheri*), Cerro Azul Volcano tortoises (*C. vicina*) and Cinco Cerros tortoises (*C. vicina/C. guntheri*) used to describe haematology and plasma biochemistry reference intervals in the Galapagos Islands.

Sampling on Alcedo Volcano occurred during a 7 day trip in July 2018, whereas in the surroundings of Sierra Negra and Cerro Azul we sampled tortoises at three different locations over 3 days in January 2020 (Fig. 1).

### Sampling design and collection

For consistency, we used the same methodology previously published by our research group (Nieto-Claudín *et al*., 2021b; 2024). In summary, we recorded morphometric measurements (curved carapace and plastron length and width with a 150 cm measuring tape) and weight (hanging scale, precision of ±0.5 kg) of each tortoise to determine the sex of mature animals and classify their age (Nieto-Claudin *et al*., 2021b). A visual examination to assess general health status was performed and up to 5 ml of blood were collected from the brachial vein using a 6 ml syringe, pre-heparinized with sodium heparin, and a 20G 1.5 inch needle. Two blood smears were made immediately at the field site, fixed using high-quality methanol and air dried (Fixative 1, JorvetTM Diff Quick Stain Kit, Jorgensen Laboratories, USA). Up to 3 ml of blood was immediately transferred to a lithium heparin tube to avoid clotting. We kept heparinized blood at 4°C until analysis within 12 h. Tortoises were identified by microchips previously placed by the Galapagos National Park Service rangers. If no microchip was detected, a new subcutaneous microchip (DATAMARS®) was placed in the caudo-ventral area of the left hind leg (Nieto-Claudin *et al*., 2021b).

### Haematology and plasma biochemistry

For haematology and plasma biochemistry analyses we used the methodology described in Nieto-Claudín *et al*., 2021b; 2024. Packed cell volume (PCV) was determined using high-speed centrifugation of blood-filled microhematocrit tubes and manual total solids (TS) were performed with a clinical refractometer (J-351, Jorgensen Laboratories, USA). Plasma was separated by high-speed centrifugation and kept frozen at –20°C until biochemistry was performed within 2 weeks of collection. Blood films were stained using the modified Wright Giemsa stain (JorvetTM Diff Quick Stain Kit, Jorgensen Laboratories, USA) following manufacturer’s instructions. We determined the estimated total white blood cell (WBC) counts using the highest quality blood film from each tortoise. White blood cell counts were read at 40× magnification on 10 different fields of the monolayer and averaged using the following equation: WBC (× 10^9^ cells/l) = (AVG 10 field on 40×)*1.6 (Sheldon *et al*., 2016; Nieto-Claudín *et al*., 2021b). WBC differential counts were performed by examining 100 WBCs on the same blood film at 100× magnification. We calculated estimated values (in percent) for heterophils, lymphocytes, monocytes, eosinophils and basophils. We determined heterophil:lymphocyte ratio (H:L) from the differential. Absolute values (ABS) for each white cell morphotype were calculated as follows: ABS WBC Diff (× 10^9^ cells/l) = [ABS WBC (× 10^9^ cells/l)] * [WBS Diff (%)] * 0.01. Two separate people (J.L.P. and M.B.W.) performed WBC estimates and differentials for this study. Counts for site Alcedo were performed by J.L.P. and those for sites Sierra Negra, Cerro Azul and Cinco Cerros were performed by M.B.W. Prior to reading blood films for this study, J.L.P. and M.B.W. trained together to calibrate WBC identification and WBC count technique and performed independent counts on the same Galapagos tortoise blood films to ensure <10% difference between observers.

We performed biochemistry analysis (Avian/Reptilian VetScan® Profile) on thawed frozen plasma, including albumin (Alb), bile acids (BA), aspartate aminotransferase (AST), calcium (Ca), creatinine kinase (CK), glucose (GLU), potassium (K), sodium (Na), phosphorus (P), globulin (Glob), total protein (TP) and uric acid (UA).

All samples were collected under the Galapagos National Park annual research permits PC-35-18 and PC-28-20 and the International Animal Care and Use Committee from the Group of Rehabilitation of Endemic Wildlife Species (GREFA-Spain) with registration number 17/001. All samples were processed and analysed at the Charles Darwin Research Station (CDRS).

### Statistical analyses

Reference Value Advisor (RefVal) v.2.1 was used as in previous manuscripts (Nieto-Claudín *et al*., 2021b; 2024) to perform descriptive statistics (mean, median, SD, min and max) and compute 95% RI and 90% confidence intervals (CI) for each analyte (Flatland *et al*., 2013). According to the ASCVP Guidelines for the Determination of Reference Intervals in Veterinary Species, non-parametric or robust methods are recommended when 40 ≤ × ≤120 reference samples are available. Although the robust method performs best when reference data have a symmetrical distribution (with or without transformation), it can be used in the absence of Gaussianity. When 20 ≤ × <40 reference samples are available, RI should be calculated by robust (distribution independent) or parametric (if Gaussianity can be established) methods. In the event of <20 samples, reference intervals should only be considered under special circumstances such as in species listed as endangered based on IUCN criteria (Flatland *et al*., 2013). Therefore, we used non-parametric methods for analytes with sample sizes ≥40, robust or parametric methods (Box–Cox transformed when appropriate) for sample sizes 20 ≤ × <40 and only descriptive statistics with sample sizes of *n* = 10–20.

The Anderson–Darling test and histogram evaluation were used to assess data symmetry and distribution. Normality tests are designed for large populations; however, in smaller population sizes it has been demonstrated that a threshold *P*-value of 0.3, instead of 0.05, more accurately characterizes the results (Flatland *et al*., 2013). For this reason, if the tests’ *P*-value was <0.3, the analyte was judged as having a non-Gaussian distribution. We identified outlier values by RefVal using Dixon and Tukey’s range tests, closely analysed each one and manually removed only those attributed to analytic error or poor sample quality.

While the Cinco Cerros location is geographically considered a part of the Cerro Azul Volcano where only *C. vicina* are found, literature supports the coexistence of *C. guntheri* and *C. vicina* tortoises at Cinco Cerros. As these species are not morphologically distinct, whether the tortoises sampled at Cinco Cerros are *C. guntheri* or *C. vicina* (or hybrids) is not definitively known. For this reason, we treated Cinco Cerros as a unique population and did not combine the data from Cinco Cerros tortoises with those from the Cerro Azul Volcano. Instead, we reported separate RI and 90% CI for the four different sampling areas (Alcedo, Sierra Negra, Cerro Azul and Cinco Cerros) and conducted statistical comparisons between these four populations.

We compared biochemistry and haematology parameters between sexes within each population if sufficient data were available. We also compared all parameters between each of the four populations sampled in this study (Alcedo, Sierra Negra, Cerro Azul and Cinco Cerros). We assessed the normality of all parameters using the Kolmogorov–Smirnov test and applied the Kruskal–Wallis (K–W) test with Bonferroni *post hoc* adjustment to test for differences.

**Table 1:** RI and CI of haematology and plasma biochemistry parameters for Alcedo Volcano adult tortoises (*C. vandenburghi*) from the Galapagos Islands. NP, nonparametric. According to ASVCP guidelines, the analyte would be judged as having a non-Gaussian (NG) distribution if the tests’ *P*-value <0.3. Packed cell volume (PCV), Total Solids/Total Proteins (TS/TP), White Blood Cell concentration (WBC conc.), and Heterophil:Lymphocyte ratio (H:L) are abbreviated

Analyte	Units	*n*	Mean	SD	Median	Min	Max	*P*-value	Distribution	Method	LRL of RI	URL of RI	90% CI of LRL	90% CI of URL	Outlier
PCV	l/l	77	23.2	2.8	23	18	28	0.003	NG	NP	18	28	18–19	27–28	1
TS/TP	g/l	76	6.9	0.7	6.9	5.6	8.4	0.198	G	NP	5.6	8.4	5.6–5.7	8.1–8.4	2
WBC conc.	10^9^/l	46	12.6	6.1	10.4	5.4	28.6	0.000	NG	NP	6	28	5.4–6.4	25–29	2
Heterophil	%	45	14.1	9.4	12	1	35	0.006	NG	NP	1	35	1.0–4	31.6–35	3
Heterophil	10^9^/l	45	1.5	1	1.6	0.1	4.5	0.203	NG	NP	0.1	4.4	0.1–0.2	3–4.5	3
Lymphocyte	%	45	75.3	14.2	79	40	94	0.008	NG	NP	41	94	40–58	93.6–94	3
Lymphocyte	10^9^/l	45	9.9	6	8.1	2.6	26.1	0.000	NG	NP	2.7	26	2.6–3.8	22–26	3
Monocyte	%	45	2.1	1.8	2	0	9	0.001	NG	NP	0	8.4	0–0	5–9	3
Monocyte	10^9^/l	45	0.2	0.2	0.2	0	0.9	0.000	NG	NP	0	0.9	0–0	0.7–0.9	3
Eosinophil	%	45	6.9	6.1	6	0	19	0.000	NG	NP	0	19	0–0.1	18.3–19	3
Eosinophil	10^9^/l	45	0.7	0.6	0.5	0	1.9	0.006	NG	NP	0	2	0–0	1.6–2	3
Basophil	%	45	1.6	1.6	1	0	8	0.000	NG	NP	0	7.6	0–0	3.9–8	3
Basophil	10^9^/l	45	0.2	0.3	0.2	0	1.9	0.000	NG	NP	0	1.7	0–0	0.5–1.9	3
H:L	%	45	0.2	0.2	0.1	0	0.9	0.000	NG	NP	0	0.9	0–0	0.5–0.9	3
Sodium	mEq/l	76	130.5	4.9	130.5	120	140	0.220	G	NP	121	140	120–123.9	140–140	1
Potassium	mEq/l	77	6.9	1.1	6.8	4.6	8.8	0.525	G	NP	4.7	8.8	4.6–5.2	8.6–8.8	0
Calcium	mg/dl	77	14.8	2.8	15.4	8.6	22.1	0.000	NG	NP	11	22	8.6–12	20–22	0
Phosphorus	mg/dl	76	3.6	0.8	3.4	2.2	6	0.002	NG	NP	2.5	5.9	2.2–2.6	4.9–6	1
Uric acid	mg/dL	76	1.6	0.8	1.4	0.5	4.5	0.000	NG	NP	0.6	4	0.5–0.8	3.4–4.5	1
Aspartate aminotransferase	U/l	73	54.8	14	53	29	92	0.407	G	NP	29	86	29–35	81–92	4
Creatinine kinase	U/l	71	897.5	595.5	790	254	3408	0.000	NG	NP	264	2526	254–303	2069–3408	6
Glucose	mg/dl	77	43.6	15.6	40	19	117	0.000	NG	NP	22	85	19–24	66–117	0
Total protein	g/dl	77	6.3	0.6	6.4	4.5	7.7	0.388	G	NP	4.9	7.7	4.5–5.4	7.2–7.7	0
Albumin	g/dl	75	1.5	0.2	1.5	1	2	0.022	NG	NP	1	2	1.0–1.2	1.9–2	2
Globulin	g/dl	75	4.8	0.5	4.8	3.3	5.8	0.491	G	NP	3.6	5.7	3.3–3.9	5.6–5.8	2

A multivariate statistical model was conducted using principal component analyses (PCA) with and without the Varimax rotation to explore differences in haematology and biochemistry parameters between tortoise populations, including those previously published by our research group (Western Santa Cruz, Eastern Santa Cruz, San Cristobal and Española) (Nieto-Claudin *et al*., 2021a; 2024). Based on the preliminary results, we applied the PCA with Varimax rotation to the same data, clustered by sex. Principal components 1 and 2 (PC1, PC2) were graphically represented. We performed the analyses in IBM® SPSS Statistics 25 and used *P* < 0.05 for all tests other than Anderson–Darling as noted above.

## Results

We sampled a total of 78 tortoises (59 females, 19 males) from Alcedo Volcano (*C. vandenburghi*), 16 tortoises (11 females, 5 males) from Sierra Negra Volcano (*C. guntheri*), 18 tortoises (5 females, 13 males) from Cerro Azul Volcano (*C. vicina*) and 25 tortoises (13 females, 12 males) from Cinco Cerros (including three individuals with ‘flat’ morphology). We calculated descriptive statistics and conducted RI and 90% CI (if sample size allowed) of haematology and plasma chemistry parameters for all four populations: Alcedo (Table 1), Sierra Negra (Table 2), Cerro Azul (Table 3) and Cinco Cerros (Table 4).

**Table 2:** Descriptive statistics of haematology and plasma biochemistry parameters for Sierra Negra Volcano adult tortoises (*C. guntheri*) from the Galapagos Islands. Following the ASVCP guidelines, reference intervals and confidence intervals were not calculated due to sample size <20. Packed cell volume (PCV), Total Solids/Total Proteins (TS/TP), White Blood Cell concentration (WBC conc.), and Heterophil:Lymphocyte ratio (H:L) are abbreviated

Analyte	Units	*n*	Mean	SD	Median	Min	Max	Outliers
PCV	l/l	14	17	3.5	18	12	22	2
TS/TP	g/l	15	5.6	1.7	4.8	3.8	9.2	1
WBC conc.	10^9^/l	12	11.2	1.9	11.2	7.2	13.6	0
Heterophil	%	12	16	5.8	15.5	5	26	0
Heterophil	10^9^/l	12	1.8	0.7	1.7	0.4	2.9	0
Lymphocyte	%	12	66.6	7.6	65.5	55	82	0
Lymphocyte	10^9^/l	12	7.4	1.4	7.2	5.2	9.8	0
Monocyte	%	12	5.8	2.4	5.5	2	10	0
Monocyte	10^9^/l	12	0.6	0.3	0.6	0.2	1.0	0
Eosinophil	%	12	3.8	2.3	3	1	8	0
Eosinophil	10^9^/l	12	0.4	0.3	0.3	0.1	0.9	0
Basophil	%	12	7.9	2.2	8.5	4	11	0
Basophil	10^9^/l	12	0.9	0.3	0.8	0.5	1.2	0
H:L	%	12	0.3	0.1	0.2	0.1	0.5	0
Sodium	mEq/l	13	136.4	4.7	137	127	144	0
Potassium	mEq/l	13	7.3	0.9	7	5.5	8.6	0
Calcium	mg/dl	11	10.4	3.9	9.1	4.9	16.1	0
Phosphorus	mg/dl	13	4.6	1.2	4.2	3.4	6.4	0
Uric acid	mg/dl	13	2	0.5	1.9	1.4	2.9	0
Aspartate aminotransferase	U/l	11	64.5	13.2	64.0	42	83	2
Creatine kinase	U/l	10	1974	1333	1733	238	4801	0
Glucose	mg/dl	13	60	11.3	57	46	83	0
Total protein	g/dl	13	4.9	0.9	4.5	3.8	6.3	0
Albumin	g/dl	13	1.4	0.5	1.2	0.9	2.2	0
Globulin	g/dl	9	3.6	0.4	3.8	3.1	4.1	0

**Table 3:** RI and CI of haematology and plasma biochemistry parameters for Cerro Azul Volcano adult tortoises (*C. vicina*) from the Galapagos Islands. P, parametric; T, transformed, if data was transformed to Gaussian prior to applying parametric or robust methods. According to ASVCP guidelines, the analyte would be judged as having a non-Gaussian (NG) distribution if the tests’ *P*-value <0.3. Packed cell volume (PCV), Total Solids/Total Proteins (TS/TP), White Blood Cell concentration (WBC conc.), and Heterophil:Lymphocyte ratio (H:L) are abbreviated

Analyte	Units	*n*	Mean	SD	Median	Min	Max	*P*-value	Distribution	Method	LRL of RI	URL of RI	90% CI of LRL	90% CI of URL	Outliers
PCV	l/l	15	21.3	3.1	22	14	26	0.207	NG	PT	12	27	3–17	25–28	1
TS/TP	g/l	15	6.6	0.9	6.4	5.4	8.8	0.141	NG	PT	5.2	9.5	4.9–5.6	8–12	1
WBC conc.	10^9^/l	15	8.2	1.8	8	5.6	12.5	0.131	NG	PT	4.8	12.5	3.5–5.9	10–14	1
Heterophil	%	15	17.3	8.2	14	8	35	0.046	NG	PT	6.8	46	5.8–8.6	30–69	1
Heterophil	10^9^/l	15	1.3	0.4	1.3	0.7	1.9	0.203	NG	PT	0.5	2.6	0.4–0.8	2–3.2	1
Lymphocyte	%	15	55.6	9.2	54	45	71	0.066	NG	PT	35.4	76	28.2–42.8	68–84	1
Lymphocyte	10^9^/l	15	4.7	1.7	4.3	2.5	8.9	0.111	NG	PT	2.3	10	2–2.8	7.2–14	1
Monocyte	%	15	6.3	2.2	6	3	10	0.241	NG	PT	2.5	12	2–3.5	9.7–15	1
Monocyte	10^9^/l	15	0.5	0.1	0.6	0.3	0.7	0.111	NG	P	0.2	0.8	0.1–0.3	0.7–0.9	1
Eosinophil	%	15	14.3	7.5	14	4	27	0.597	G	PT	1.7	35	0.2–4.8	26–46	1
Eosinophil	10^9^/l	15	1.2	0.6	1	0.3	2.3	0.222	NG	PT	0	2.6	0–0.3	2.1–3.2	1
Basophil	%	15	6.5	2.1	7	3	11	0.778	G	PT	2	11	0.3–3.6	9.6–13	1
Basophil	10^9^/l	15	0.5	0.2	0.5	0.2	0.9	0.248	NG	P	0	1.1	0–0.2	0.9–1.2	1
H:L	%	15	0.3	0.2	0.3	0.1	0.8	0.030	NG	PT	0.1	0.9	0–0.1	0.6–1.2	1
Sodium	mEq/l	15	131	2.4	130	127	135	0.505	G	PT	125	136	123–127	134–138	0
Potassium	mEq/l	15	5.8	0.8	5.9	4.1	6.9	0.875	G	PT	3.6	7.2	2.2–4.6	6.8–7.6	0
Calcium	mg/dl	16	12.7	2.4	11.5	10.1	116.1	0.000	NG	P	7.5	18	7–8	16–19	0
Phosphorus	mg/dl	15	3.5	0.4	3.4	2.8	4.3	0.709	G	PT	2.7	4.5	2.5–3	4.1–4.9	0
Uric acid	mg/dl	16	1	0.2	1.1	0.6	1.3	0.100	NG	P	0.5	1.5	0.4–0.7	1.3–1.6	0
Aspartate aminotransferase	U/l	14	34.8	7.8	33	24	54	0.079	NG	P	17	52	12–24	46–59	0
Creatine kinase	U/l	14	961.8	510.5	869.5	381	2367	0.008	NG	PT	272	2338	194–419	1658–3159	0
Glucose	mg/dl	16	44.4	10.7	41	32	67	0.076	NG	PT	29	80	27–33	61–115	0
Total protein	g/dl	15	6	0.5	6	5	6.5	0.216	NG	P	5	7	4.6–5.3	6.6–7.4	0
Albumin	g/dl	15	1.4	0.3	1.3	1.1	1.9	0.119	NG	PT	1	2.1	1–1.1	1.8–2.6	0
Globulin	g/dl	15	4.6	0.5	4.6	3.8	5.3	0.298	NG	PT	3.3	5.7	2.6–3.8	5.3–6	0

**Table 4:** RI and CI of haematology and plasma biochemistry parameters for Cinco Cerros adult tortoises (*C. vicina/C. guntheri*) from the Galapagos Islands. P, parametric; R, robust; add T, transformed, if data was transformed to Gaussian prior to applying parametric or robust methods. According to ASVCP guidelines, the analyte would be judged as having a non-Gaussian (NG) distribution if the tests’ *P*-value <0.3. Packed cell volume (PCV), Total Solids/Total Proteins (TS/TP), White Blood Cell concentration (WBC conc.), and Heterophil:Lymphocyte ratio (H:L) are abbreviated

Analyte	Units	*n*	Mean	SD	Median	Min	Max	*P*-value	Distribution	Method	LRL of RI	URL of RI	90% CI of LRL	90% CI of URL	Outliers
PCV	l/l	23	19.0	1.8	18	16	22	0.003	NG	RT	15	23	14.5–16.3	22–24	1
TS/TP	g/l	22	6.3	0.8	6.3	4.8	7.8	0.482	G	PT	4.6	8.1	4.2–5.1	7.6–8.7	2
WBC conc.	10^9^/l	23	8.1	7.7	1.7	5.8	11.7	0.140	NG	RT	5.3	13	5–5.9	11–15	1
Heterophil	%	20	14.3	7.3	15	5	28	0.015	NG	RT	3.1	39.5	1.6–6	26–54	3
Heterophil	10^9^/l	20	1.2	0.6	1.1	0.4	2.4	0.123	NG	PT	0	2.4	0–0.1	1.9–2.9	3
Lymphocyte	%	20	56.6	56.5	10	43	78	0.353	G	RT	39	84	37–44	74–94	3
Lymphocyte	10^9^/l	20	4.8	1.6	4.3	2.6	8.1	0.120	NG	RT	2.4	9.3	2.1–2.8	7.2–11.5	3
Monocyte	%	20	6.1	3	6	1	11	0.433	G	RT	0	12.5	0–1.4	10.4–14.3	3
Monocyte	10^9^/l	20	0.5	0.2	0.6	0.1	0.9	0.118	NG	PT	0	1.1	0–0.2	0.9–1.2	3
Eosinophil	%	20	16.4	7	16.5	5	32	0.893	G	RT	3.3	33	1.2–7	28–39	3
Eosinophil	10^9^/l	20	1.3	0.6	1.1	0.4	3	0.075	NG	PT	0.4	2.9	0.2–0.6	2.3–3.7	3
Basophil	%	20	6.8	2.8	6.5	2	12	0.277	NG	R	0.4	13	0–2.2	10.6–14.5	3
Basophil	10^9^/l	20	0.6	0.2	0.5	0.1	1.1	0.590	G	RT	0.1	1.1	0.1–0.2	0.9–1.4	3
H:L	%	20	0.3	0.2	0.3	0.1	0.6	0.004	NG	RT	0	0.9	0–0.1	0.5–1.5	3
Sodium	mEq/l	20	133.1	3.4	133.5	124	138	0.383	G	RT	126	141	123–129	138–143	1
Potassium	mEq/l	20	6.8	0.9	6.8	5.4	8.6	0.224	NG	RT	5	9	4.6–5.5	8.2–10	0
Calcium	mg/dl	21	12.3	2.6	11.9	7.9	16.1	0.053	NG	RT	7.5	19	6.5–8.6	16–21	0
Phosphorus	mg/dl	21	3.7	0.7	3.6	2.8	5.4	0.005	NG	PT	2.7	6.1	2.6–2.9	4.8–8.3	0
Uric acid	mg/dl	21	1.3	0.4	1.2	0.7	2	0.014	NG	PT	0.7	2.2	0.6–0.8	1.8–2.5	0
Aspartate aminotransferase	U/l	20	38.4	10.5	36	22	60	0.131	NG	RT	22	68	19–25	55–82	1
Creatine kinase	U/l	18	968	592.7	718	325	2281	0.007	NG	PT	286	3034	243–370	1940–4603	0
Glucose	mg/dl	21	38.3	7.6	38	25	55	0.579	G	RT	24	56	21–28	50–62	0
Total protein	g/dl	20	5.8	0.5	5.8	4.7	6.6	0.486	G	RT	4.3	6.8	3.8–4.8	6.5–7	1
Albumin	g/dl	21	1.2	0.3	1.2	0.9	1.9	0.007	NG	PT	0.6	1.9	0.5–0.7	1.7–2.1	1
Globulin	g/dL	14	4.5	0.5	4.5	3.6	5.4	0.827	G	PT	3.3	5.6	2.9–3.8	5.2–6.1	0

**Figure 2:**
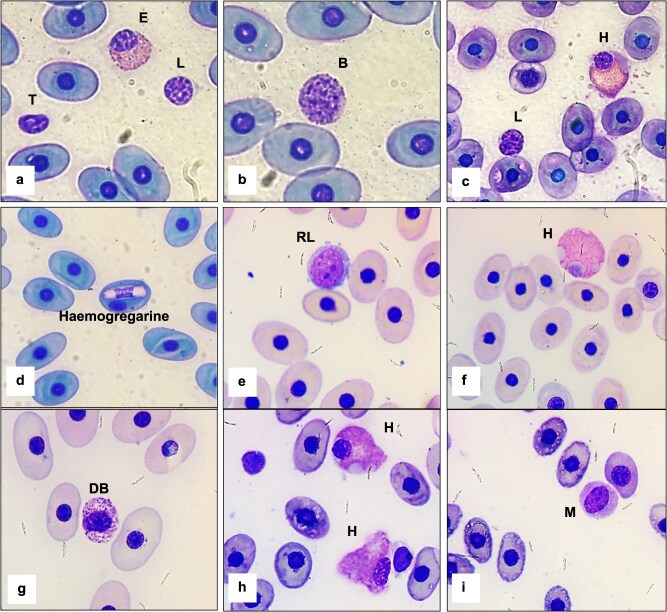
Modified Wright–Giemsa-stained peripheral blood from Alcedo Volcano (*C. vandenburghi*) (a–d), Cerro Azul Volcano (*C. vicina*) (e–f), Cinco Cerros (*C. vicina/C. guntheri*) (g) and Sierra Negra Volcano giant tortoises (*C. guntheri*) (h–i). L, lymphocyte; RL, reactive lymphocyte; T, thrombocyte; H, heterophil; B, basophil; DB, degranulated basophil; M, monocyte; E, eosinophil. Erythrocyte from Alcedo Volcano giant tortoises with potential hemogregarine (d).

Morphological characteristics of the WBCs are shown in Fig. 2. Drying artefact derived from extreme field conditions and slow dry time was present across slides and evident in erythrocytes. No significant differences in cell morphology were observed in comparison with those previously described in other Galapagos tortoise species (Nieto-Claudin *et al*, 2021b; 2024). Basophils were degranulated in some smears. No cell toxicity was identified, and thrombocytes were qualitatively adequate across smears although thrombocyte and red blood cell counts were not performed. Organisms compatible with hemogregarines were observed in the red blood cells of two blood smears from Alcedo Volcano tortoises with an abundance of 1/100 RBCs on average (Fig. 2).

Comparing haematology parameters between the four Isabela locations, we found that tortoises from Sierra Negra showed significantly lower PCV and TS than the other three locations (*P* < 0.001). Tortoises from Cerro Azul and Cinco Cerros had significantly lower WBC counts than those from Alcedo and Sierra Negra (*P* < 0.001). No differences were observed in heterophils, whereas lymphocytes were significantly higher in Alcedo tortoises and monocytes and basophils significantly lower, when compared to tortoises from the other three locations (*P* < 0.001). Eosinophils were significantly lower in tortoises from Alcedo and Sierra Negra, when compared to Cerro Azul and Cinco Cerros (*P* < 0.001). No differences were found in H:L ratio (Table 5).

**Table 5:** RI summary of haematology and plasma biochemistry parameters for free-living Galapagos adult tortoises from this and previous studies conducted by our research group (Nieto-Claudín *et al*., 2021b and 2024). Packed cell volume (PCV), Total Solids/Total Proteins (TS/TP), White Blood Cell concentration (WBC conc.), and Heterophil:Lymphocyte ratio (H:L) are abbreviated. *Reference intervals were not calculated due to sample size <20; maximun and minimum values are included instead.

Measurand	Units	RI by tortoise species/location							
		Alcedo Volcano (*C. vandenburghi*)	Cerro Azul (*C. vicina*)	Cinco Cerros (*C. vicina/C. guntheri*)	Sierra Negra^*^ (*C. guntheri*)	Eastern Santa Cruz (*C. donfaustoi*)	San Cristobal (*C. chathamensis*)	Española (*C. hoodensis*)	Western Santa Cruz (*C. porteri*)
PCV	l/l	18–28	12–27	15–23	12–22	14–31	10–27	15–30	14–26
TS/TP	g/l	5.6–8.4	5.2–9.5	4.6–8	3.8–9.2	3.6–9	3.4–7.7	3.9–6.8	4.6–10
WBC conc.	10^9^/l	5.6–28	4.8–12	5.3–13	7.2–14	4.8–17	4.8–14	2.1–19	6.6–40
Heterophil	%	1–35	7–46	3–39	5–26	8–39	10–38	4–28	2–30
Heterophil	10^9^/l	0.1–4.4	0.5–2.6	0–2.4	0.4–2.9	0.5–4.7	0.8–3.7	0.2–2.4	0.3–5.3
Lymphocyte	%	41–94	35–76	40–84	55–82	45–79	48–78	51–91	55–95
Lymphocyte	10^9^/l	2.7–26	2.3–10	2.4–9.3	5.2–10	2.3–11	3.4–11	1.4–18	4.5–36
Monocyte	%	0–8.4	2.5–12	0–12.5	2.0–10	0–13	2.3–12	0–6.9	0–5.0
Monocyte	10^9^/l	0–0.9	0.2–0.8	0–1.1	0.2–1.0	0–1.4	0.1–1.1	0–0.6	0–1.2
Eosinophil	%	0–19	1.7–35	3.3–33	1.0–8.0	0–8	0–24	0–1.9	0–17
Eosinophil	10^9^/l	0–2	0–2.6	0.4–2.9	0.1–0.9	0–0.1	0–0.2	0–0.1	0–2.2
Basophil	%	0–8	1.9–11	0.4–13	4.4–11	0–16	0–13	0–17	0–10
Basophil	10^9^/l	0–1.7	0–1.1	0.1–1.1	0.5–1.2	0–1.3	0–1.1	0–2.0	0–2.1
H:L	%	0–0.9	0.1–0.9	0–0.9	0.1–0.5	0.1–0.9	0.1–0.7	0.1–0.5	0–0.5
Sodium	mEq/l	121–140	125–134	126–141	127–144	124–141	125–135	120–140	117–138
Potassium	mEq/l	4.7–8.8	3.6–7.2	5.1–9	5.5–8.6	5.8–8.6	4.2–8.8	4.7–9.1	4.1–8.6
Calcium	mg/dl	11–22	7.5–18	7.5–19	4.9–16	8.1–20	5.3–17	4.5–15	7.8–20
Phosphorus	mg/dl	2.5–5.9	2.7–4.5	2.7–6.1	3.4–6.4	2.4–5.4	2.3–5.4	2.14.9	3.2–5.4
Uric acid	mg/dl	0.6–4	0.5–1.5	0.7–2.2	1.4–2.9	0.4–3.2	0.7–3.2	0.8–2.6	0.8–3.2
Aspartate aminotransferase	U/l	29–86	17–52	22–68	42–83	24–92	20–71	31–114	25–84
Creatine kinase	U/l	264–2526	272–2338	286–3034	238–4801	272–3828	221–4000	298–7675	260–5296
Glucose	mg/dl	22–85	29–80	24–56	46–83	23–120	25–98	23–76	27–123
Total protein	g/dl	4.9–7.7	5.0–7.0	4.3–6.8	3.8–6.3	2.9–7.6	2.5–5.8	3.4–5.9	3.7–7.7
Albumin	g/dl	1.0–2.0	1–2.1	0.6–1.9	0.9–2.2	0.4–2.4	1–2.2	1.1–2.3	1.1–2.5
Globulin	g/dl	3.6–5.7	3.3–5.7	3.3–5.6	3.1–4.1	2.3–5	2.4–4.1	2.2–4	2.9–5.9

Biochemistry parameters showed some differences between species, with Sierra Negra tortoises presenting significantly higher CK, UA, Glu, P and Na, and significantly lower TP, Alb and Glob than the other three populations (*P* < 0.001). Cerro Azul tortoises showed significantly lower AST, UA and K when compared to the other three populations (*P* < 0.001). Samples from Alcedo were significantly less hemolyzed and lipemic than the other three populations (*P* < 0.001); Sierra Negra samples were the most hemolyzed and Cerro Azul samples the most lipemic (*P* < 0.001) (Table 5).

Comparisons between sexes showed significantly higher values of calcium in females than males from all locations (*P* < 0.001). In Cerro Azul and Sierra Negra, females also presented significantly higher *P* and samples were more lipemic when compared to males of the same species (*P* < 0.01). In Alcedo Volcano, males showed significantly higher PCV, Na and eosinophils (*P* < 0.01), whereas females had higher AST, UA, P and monocytes (*P* < 0.001) than males of the same species.

**Figure 3:**
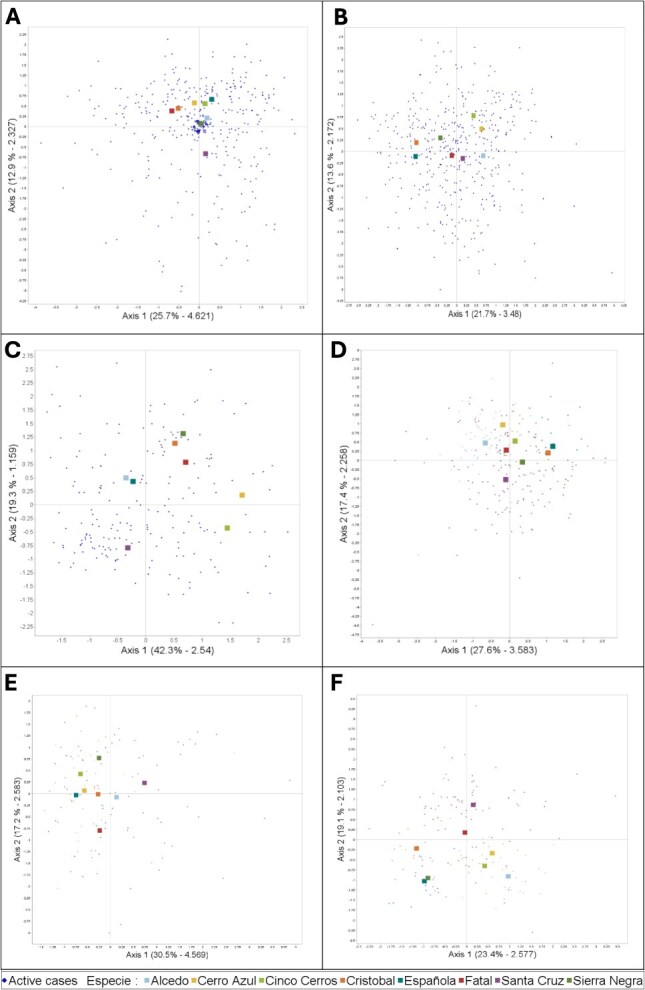
Graphical representation of Principal Components 1 (Axis 1) and 2 (Axis 2) with varimax rotation of Galapagos giant tortoise haematology and biochemistry parameters from eight different populations: (a) haematology parameters of all individuals; (b) biochemistry parameters of all individuals; (c) haematology parameters of females; (d) biochemistry parameters of females; (e) haematology parameters of males; and (f) biochemistry parameters of males. Haematological variables represented in (a), (c), and (e) are heterophils, lymphocytes, absolute white blood cell counts, and H:L ratio. Blood chemistry variables represented in (b) are total solids, total proteins, albumin, globulin and calcium. Variables represented in (d) are total solids, total proteins, calcium, globulin, uric acid and glucose. Variables represented in (f) are glucose, phosphorus, total proteins, globulin and sodium.

Statistical models obtained using PCA with Varimax rotation identified six main principal components (PC) which explained a cumulative variance of 80.1% of haematology parameters and 71.5% of plasma chemistry parameters. A 40.1% of the cumulative variance of PC1 and PC2 would be explained by four hematologic variables: heterophils, lymphocytes, ABS WBC and H:L ratio; whereas the five blood chemistry variables more correlated to PC1 and PC2 were TS, TP, Alb, Glob, and Ca, representing a 37.4 of cumulative variance (Fig. 3). Additional comparisons clustered by sex showed similar results between males and females, as described above, for haematology variables. However, differences were observed when comparing blood chemistry variables clustered by sex. In males, 42.5% of the cumulative variation of PC1 and PC2 would be explained by Glu, P, TP, Glob and Na, whereas in females, 44.9% of the cumulative variation was explained by TS, TP, Ca, Glob, UA and Glu parameters (Fig. 3). Western Santa Cruz values did not cluster with any of the other seven tortoise populations. Table 5 summarizes haematology and biochemistry RI for all eight tortoise species and populations described in this and previous manuscripts (Nieto-Claudin *et al*., 2021b; 2024).

## Discussion

This study constitutes the first description of haematology and biochemistry RI in four tortoise populations from southern Isabela encompassing three different species (*C. guntheri, C. vicina,* and *C. vandenburghi*). Moreover, we provide morphologic characteristics of WBCs and compared blood values of this study with data of 437 free-living tortoises of seven different species from the main islands of the archipelago: Isabela, Santa Cruz, San Cristobal and Española.

Sex-based differences found in some haematological and chemistry parameters are consistent with findings in other Galapagos tortoise species (Nieto-Claudin *et al*., 2021b; 2024) and turtles around the world (Heatley and Russell, 2019). Overall, females had higher Ca, P and Alb, as is consistent with vitellogenesis, whereas males had higher PCV and Na than females. Differences in AST, UA, monocytes and eosinophils may be explained by differences in seasonality, reproductive status, and small sample size, such as for the Sierra Negra tortoises, which were hard to locate and thus, we sampled fewer animals using the same sampling effort.

As we expand our knowledge of reptile physiology, including haematology and biochemistry reference values, we better understand how several factors may influence blood values, including inter- and intraspecific variations, sex, seasonality, habitat, movement ecology, fitness and health status, climate and anthropogenic impacts such as toxins and pollutants (Stacy *et al*., 2011; Scope *et al*., 2013; Blake *et al*., 2015; Heatley and Russell, 2019). These and other contributing factors could justify why PCA models did not explain >50% of the variance in haematology and biochemistry values across tortoise populations and sex. Cinco Cerros (the sampling area where two different species of tortoises may coexist) clustered closer to Cerro Azul Volcano than to Sierra Negra Volcano, which may indicate a higher proportion of *C. vicina* in the Cinco Cerros sample, but the relatively small sample size of these locations supports further research is warranted. Interestingly, western Santa Cruz values did not cluster with any of the other seven tortoise populations in any of the PCA graphical representations, which could be explained by the significantly higher sample size (>160 individuals) when compared to the other areas, together with sampling across multiple seasons and habitats (i.e. humid highlands and dry lowlands). While researchers continue applying novel genetic tools to defend the coexistence of several Galapagos tortoise species (Poulakakis *et al*., 2020; Jensen *et al*., 2022; Gaughran *et al*., 2024) versus one single ancestor with multiple subspecies (Kehlmaier et al., 2021), it is unknown whether these genetic differences may truly result in different haematology and plasma chemistry values across tortoise species or subspecies. More complex statistical models incorporating additional factors (i.e. seasonality, movement strategy, reproductive status) may better elucidate the presence or lack of true differences in blood values across species, subspecies and populations.

Blood cell morphology in free-living Galapagos tortoises has been described in *C. porteri, C. hoodensis, C. chathamensis* and *C. donfaustoi* (Nieto-Claudin *et al*., 2021b; 2024). The current study provides insights into three additional Galapagos tortoise species concluding that cell morphology is similar across species and provides visual resources that may be used by clinicians and researchers for future diagnostics and conservation efforts. Intracellular blood parasites compatible with hemogregarines were only observed in a few Alcedo Volcano giant tortoise (*C. vandenburghi*) smears. *Argas (Microargas) transversus* and *Amblyomma* spp. ticks have been described in giant tortoises from Isabela Island; however, their role as potential vectors for hemoparasites such as hemogregarines is still unclear (Flanagan, 2021). In Galapagos marine iguanas, hemoparasites from the genera *Hepatozoon* and/or *Hemolivia* (Apicomplexa: Eucoccidiorida) have been described, with a prevalence of up to 50% in some populations (Scheibel *et al.*, 2022). In Galapagos land iguanas, tick-transmitted apicomplexan hemoparasites of the genus *Hepatozoon* have also been described but correlation between infection and iguana fitness and well-being remains poorly understood (Onorati *et al.*, 2017).

The current study contributes to our understanding of Galapagos tortoise health by providing baseline information that can inform diagnostics and management decisions of free-living Galapagos tortoises, as well as those under human care. In Galapagos, the National Park Directorate has implemented annual health assessments of tortoises maintained within their facilities, based on the results obtained from this and previous studies conducted by the GTMEP (Nieto-Claudin *et al*., 2021b; 2024). Reference values described for the different tortoise species are being used on a regular basis at all three of the tortoise captive-breeding centres in Galapagos. They are also useful to evaluate and monitor the health of giant tortoises confiscated from the illegal trade. Moreover, through our research group, we have trained many local veterinarians and researchers to conduct full health assessments and perform and interpret manual haematology and plasma biochemistry in-country.

In conclusion, this work constitutes additional efforts to provide tortoise health baseline information that can inform conservation and management decisions in Galapagos. As the world faces new challenges and threats to protect unique and fragile environments like those in the Galapagos archipelago, understanding the health status of endemic and iconic species is mandatory to inform current and future conservation priorities and actions. We recommend that additional research focused on understanding how intraspecific and anthropogenic factors (i.e. climate change, pesticides, plastics) may affect blood parameters, and overall health, in Galapagos tortoises would contribute to a deeper understanding of their physiology and ultimately improve diagnostics and management actions to preserve these unique species.

## Data Availability

The data underlying this article will be shared upon reasonable request to the corresponding author.
